# Renoprotective Effect of *Chrysanthemum coronarium* L. Extract on Adenine-Induced Chronic Kidney Disease in Mice

**DOI:** 10.3390/ph16071048

**Published:** 2023-07-24

**Authors:** Yi-Seul Kim, Ae-Sin Lee, Haeng-Jeon Hur, Sang-Hee Lee, Hyun-Jin Na, Mi-Jeong Sung

**Affiliations:** Research Group of Natural Materials and Metabolism, Food Functionality Research, Korea Food Research Institute, 245 Nongsaenmyeong-ro, Iseo-myeon, Wanju-gun 55365, Jeollabuk-do, Republic of Korea

**Keywords:** *Chrysanthemum coronarium* L. extract, inflammation, fibrosis, chronic kidney disease

## Abstract

Chronic kidney disease (CKD) gradually leads to loss of renal function and is associated with inflammation and fibrosis. *Chrysanthemum coronarium* L., a leafy vegetable, possesses various beneficial properties, including anti-oxidative, anti-inflammatory, and antiproliferative effects. In this study, we investigated the renoprotective effect of *Chrysanthemum coronarium* L. extract (CC) on adenine (AD)-induced CKD in mice. CKD was induced by feeding mice with an AD diet (0.25% *w*/*w*) for 4 weeks. Changes in renal function, histopathology, inflammation, and renal interstitial fibrosis were analyzed. The adenine-fed mice were characterized by increased blood urea nitrogen, serum creatinine, and histological changes, including inflammation and fibrosis; however, these changes were significantly restored by treatment with CC. Additionally, CC inhibited the expression of the inflammatory markers, monocyte chemoattractant protein-1, interleukins-6 and -1β, intercellular adhesion molecule-1, and cyclooxygenase 2. Moreover, CC suppressed the expression of the fibrotic markers, type IV collagen, and fibronectin. Furthermore, CC attenuated the expression of profibrotic genes (tumor growth factor-β and α-smooth muscle actin) in AD-induced renal injury mice. Thus, our results suggest that CC has the potential to attenuate AD-induced renal injury and might offer a new option as a renoprotective agent or functional food supplement to manage CKD.

## 1. Introduction

Chronic kidney disease (CKD) is a global health challenge in both developed and underdeveloped countries. It is characterized by a progressive and irreversible loss of kidney function, with the rate of functional decline varying based on the disease severity and patient co-morbidity [1]. When CKD progresses to end-stage kidney failure, dialysis and transplantation are needed, which usually result in a decline in functional capacity and a loss of personal independence, and places a burden on health and societal support systems as well as economic burden on family and society [2,3]. Moreover, CKD seriously worsens the health conditions of patients and decreases their quality of life. Although intensive efforts have been made to discover efficient treatments that target the progression of tubulointerstitial fibrosis, there are no therapeutic agents for several types of CKD. Currently, alternatives to manage CKD rely on hypertension control through the inhibition of the renin–angiotensin system [4]. Additionally, although previous studies have focused on developing new and efficient treatments, effective agents for preventing or delaying the development of CKD remain limited [5]. Therefore, identifying an effective agent that can satisfactorily treat CKD is crucial.

There is evidence that oxidative stress and inflammation are involved in the pathophysiology of CKD [6]. Renal fibrosis, the most common feature of CKD, is characterized by the infiltration of inflammatory cells, injury to renal tubules, and tubulointerstitial fibrosis [7]. These features are observed in both humans and animals [8]. Some animal models of CKD are commonly used to confirm the progression of the disease and identify effective therapies. Adenine (AD), which is metabolized to form 2,8 dihydroxyadenine, induces renal damage via the accumulation of its metabolite. AD-induced CKD in rodents is a common technique for mimicking the functional abnormality in patients with renal failure [9,10].

Several studies have documented the beneficial effects of herbs, edible medicinal products, and traditional foods on CKD [11,12]. Several edible medicinal foods reportedly have renoprotective effects on AD-induced CKD. These edible natural products contain many bioactive components that can act on multiple targets and exert various protective effects against CKD. For example, the ErHuang Formula exhibits renoprotective effects via suppressing inflammatory and fibrotic effects [3]. *Garcinia lucida* Vesque (Clusiaceae), which contains gallic acid and quercetin, ameliorates renal damage by exerting anti-oxidant effects [4]; *Laminaria japonica*, which contains polysaccharides, improves chronic renal failure [13]; *Lindera aggregate*, which mainly contains furan sesquiterpenes, lactones, volatile oils, flavonoids, and isoquinoline alkaloids, had renoprotective effects on CKD [14]; and *Salvia miltiorrhiza*, which contains salvianolic acids and tanshinones, exhibited renoprotective effects by regulating the metabolic profile [15]. Based on these results, many researchers, including us, have focused on in-depth studies of edible natural products to identify agents for protecting against or delaying CKD progression.

*Glebionis coronaria* (*Chrysanthemum coronarium* L.) is widely distributed in the Mediterranean region. It is considered a healthy food in Asia. It is also known as Ssukgat in Korea and is a precious vegetable and medicinal plant [16,17]. Studies have shown that *Chrysanthemum coronarium* L. extract (CC) exerts potent antioxidant effects [18,19,20]. Similarly, we previously found the preventive effects of CC on bone metabolism in vitro and in vivo [16]. In addition, we recently showed that CC protects against premature senescence in vitro [17]. Based on these results, it is likely that the beneficial effects of CC are due to the complex interaction of its multiple compounds. We previously analyzed the components of CC and found that it contains several compounds, such as rutin, chlorogenic acid, cynarine, 3,4-dicaffeoylquinic acid, and 3,5-dicaffeoyl-succinoylquinic acid [14,15]. Although CC and CC-derived compounds have been shown to possess various activities, their role in CKD is unknown. Hence, in the present study, we aimed to determine whether CC exerted renoprotective effects against AD-induced renal injury.

## 2. Results

### 2.1. Ultraperformance Liquid Chromatography (UPLC)-Quadrupole Time-of-Flight Mass Spectrometry (QTOF/MS) Analysis of CC

**Figure 1:**
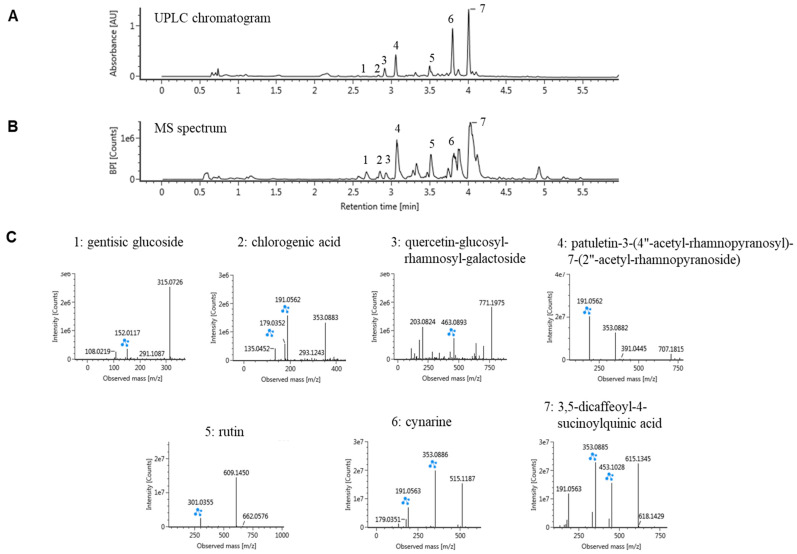
Qualitative analysis of bioactive compounds from CC. The sample extract was analyzed using UPLC equipped with an Acquity BEH C18 column (2.1 × 100 mm, 1.7 μm, Waters) at 254 nm (**A**), and the eluted compounds were ionized by negative electrospray ionization and analyzed using Q-TOF (**B**). The compounds were identified using their MS spectra (**C**) and the online database connected to the UNIFI 1.9.2.045 software. Blue symbols are fragment.

The UPLC analysis revealed several peaks at 254 nm for CC at retention times, indicating the presence of several compounds (Figure 1A). The CC was then subjected to UPLC-QTOF/MS in the negative ion mode. The base peak intensity chromatograms and their chemical structures are presented (Figure 1B). Putative metabolite identification was conducted through a database search of accurate mass and MS fragmentation patterns. The following compounds were identified as constituents of CC: gentisic glucoside, chlorogenic acid, quercetin-glucosyl-rhamnosyl-galactoside, patuletin-3-(4″-acetyl-rhamnopyranosyl)-7-(2″-acetyl-rhamnopyranoside), rutin, cynarine, and 3,5-dicaffeoyl-4-succinoylquinic acid (Figure 1C).

### 2.2. CC Attenuates AD-Induced Renal Injury

**Figure 2:**
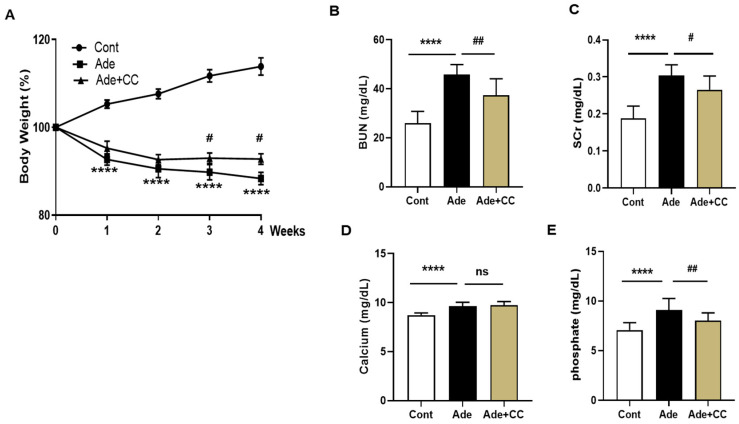
CC attenuates adenine (AD)-induced renal injury. Chronic kidney disease (CKD) was induced by AD (0.25% *w*/*w*) for 28 days, and CC (100 mg/kg body weight) was administered orally by gavage either alone or with AD. (**A**) Body weight. (**B**) BUN level. (**C**) SCr level. (**D**) Calcium level. (**E**) Phosphate level. All data are presented as mean ± standard deviation (SD). *n* = 8. **** *p* < 0.0001 vs. Cont group. ^#^ *p* < 0.05, and ^##^ *p* < 0.01, ns: not significant, vs. Ade group.

**Figure 3:**
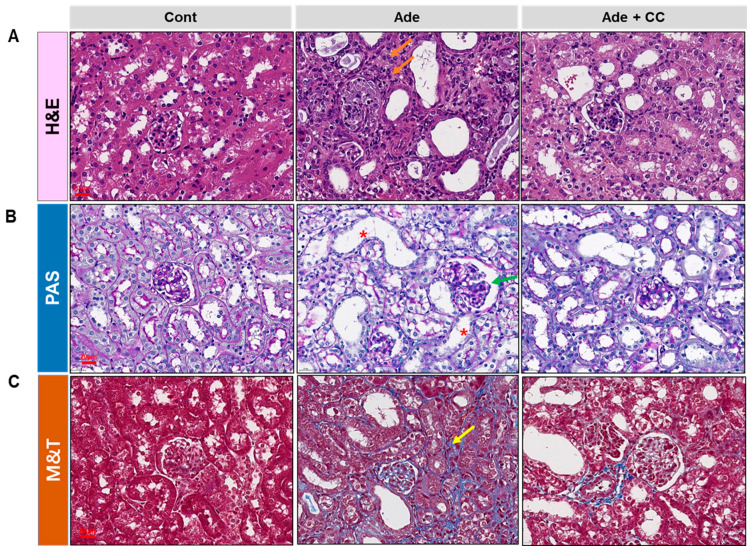

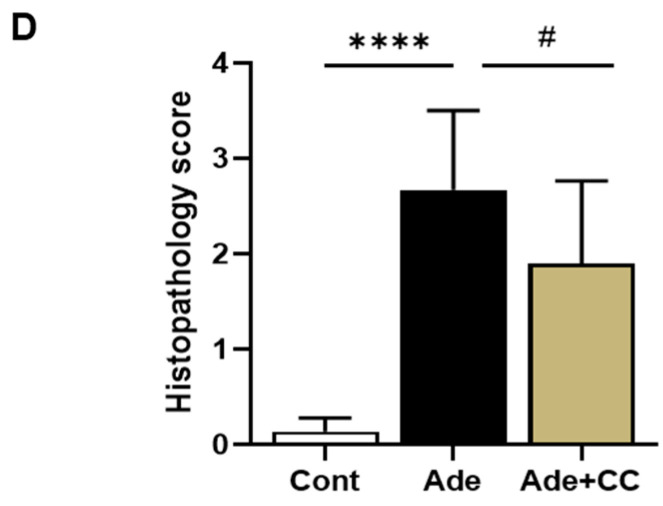
CC attenuates AD-induced morphological changes. (**A**) H&E staining was used for identification and semiquantitative scoring of inflammation. The orange arrows indicate inflammatory cell infiltration. (**B**) PAS staining was used for identification and semiquantitative scoring of basal membrane atrophy and dilatation. Green arrows indicate examples of atrophic basal membranes and red stars indicate tubule dilation. (**C**) MT staining was used for identification and semiquantitative fibrosis scoring. The yellow arrows indicate interstitial fibrosis. (**D**) Histopathology was evaluated from the kidney stained with HE, PAS, and MT. All data are presented as mean ± SD. *n* = 8. **** *p* < 0.0001 vs. control group. ^#^ *p* < 0.05, vs. Ade group.

Mice were fed an AD diet for 4 weeks to establish a CKD model. The body weight (BW) in the Cont group increased; however, it decreased in the Ade group. Although the BW of mice in the Ade+CC group was decreased, it was higher than that in the Ade group (Figure 2A). Blood urea nitrogen (BUN) and serum creatinine (SCr) levels are known indicators of kidney function. Next, the levels of BUN and SCr in the Ade group were significantly increased compared with those in the Cont group (*p* < 0.0001 for both). However, compared with those in the Ade group, BUN and SCr levels in the Ade+CC group were reduced (*p* < 0.01, *p* < 0.05, respectively). CKD is associated with an imbalance in calcium and phosphorus homeostasis (Figure 2B,C). The levels of calcium and phosphorus in the Ade group were significantly increased compared with those in the Cont group (*p* < 0.0001, for both). However, compared with those in the Ade group, the levels of calcium and phosphorus in the Ade+CC group were decreased (ns and *p* < 0.01, respectively) (Figure 2D,E). Hematoxylin and eosin (H&E), periodic acid–Schiff (PAS), and Masson’s trichrome (MT) staining were performed on the kidney sections from each group to investigate the effects of CC on histopathological changes. As shown in Figure 3A,B,D, there were no specific changes in the Cont group. However, renal damage, evidenced by inflammatory cell infiltration, renal tubular dilation, and atrophic basal membranes, was observed in the Ade group. Nonetheless, the renal damage was significantly reduced in the Ade+CC group. As shown in Figure 3C,D, the fibrosis in the Ade group was significantly increased compared with that in the Cont group. However, the fibrosis was significantly reduced in the Ade+CC group. These results suggest that CC could improve renal function and suppress renal damage in CKD mice.

### 2.3. CC Suppresses Inflammatory Responses in AD-Induced Renal Injury Mice

**Figure 4:**
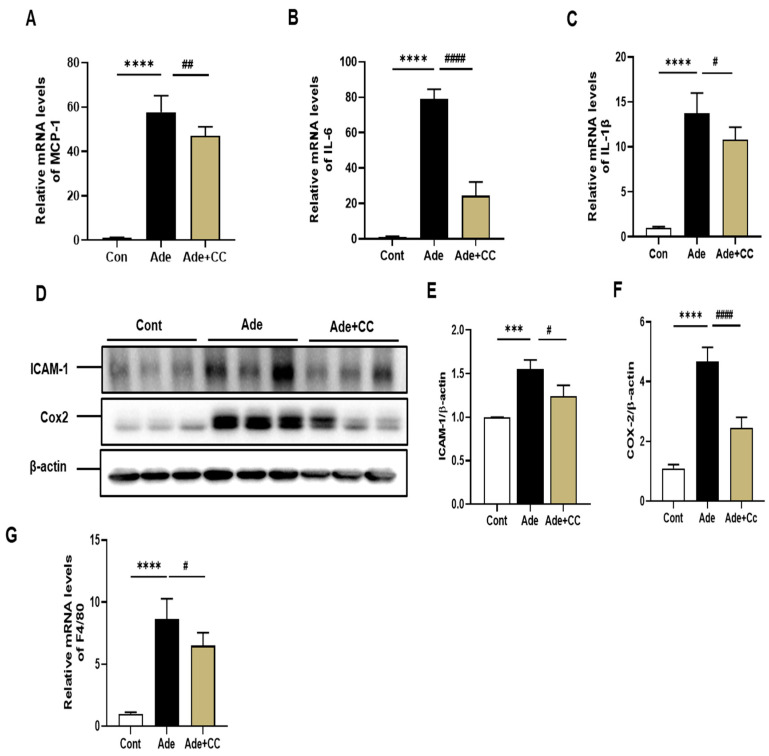
CC suppresses inflammatory responses in AD-induced renal injury mice. qRT-PCR analysis of MCP-1 (**A**), IL-6 (**B**), and IL-1β (**C**) mRNA expression. Western blotting analysis showing protein expression of ICAM-1 (**D**,**E**) and COX-2(**D**,**F**). qRT-PCR analysis showing the mRNA expression of F4/80 (**G**). All data are presented as mean ± SD. *n* = 8. *** *p* < 0.001 and **** *p* < 0.0001 vs. Cont group. ^#^ *p* < 0.05, and ^##^ *p* < 0.01, ^####^ *p* < 0.0001, vs. Ade group.

There is evidence that chronic inflammation accelerates the progression of fibrosis [6]. To define the effect of CC in regulating inflammation, we analyzed the expression of inflammatory cytokines, including monocyte chemoattractant protein (MCP)-1, interleukins (IL)-6 and IL-1β, and intercellular adhesion molecule (ICAM)-1, cyclooxygenase (COX)-2, and macrophage activation markers F4/80. Quantitative real-time polymerase chain reaction (qRT-PCR) data indicated that MCP-1, IL-6, and IL-1β levels were markedly increased in the Ade group compared with those in the Cont group (*p* < 0.0001, for all), which were decreased in the Ade+CC group (*p* < 0.01, *p* < 0.001, and *p* < 0.05, respectively; Figure 4A–C). In addition, Western blotting analysis showed that the expression levels of ICAM-1 and COX-2 were increased in the Ade group compared with those in the Cont group (Figure 4D–F, *p* < 0.0001) and were decreased in the Ade+CC group (*p* < 0.05). Moreover, qRT-PCR results revealed that the expression level of F4/80 was increased in the Ade group compared with that in the Cont group (Figure 4G, *p* < 0.0001), which was decreased by CC treatment (*p* < 0.05). These data indicate that CC attenuated inflammation in AD-induced renal injury by suppressing inflammatory cytokine production and macrophage infiltration.

### 2.4. CC Suppresses Extracellular Matrix (ECM) Deposition and Tubulointerstitial Fibrosis in AD-Induced Renal Injury Mice

**Figure 5:**
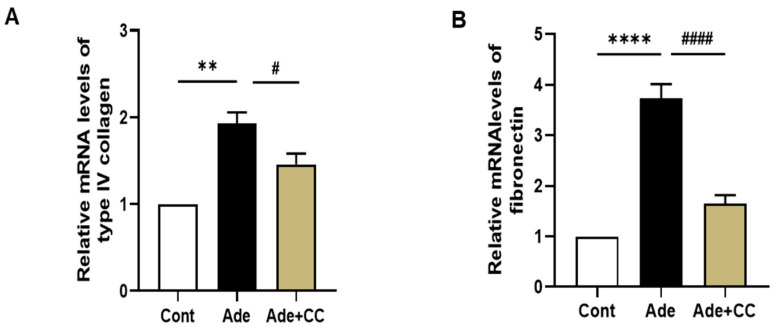
CC reduces extracellular matrix deposition and tubulointerstitial fibrosis in AD-induced renal injury mice. qRT-PCR data showing the mRNA level of type IV collagen (**A**) and fibronectin (**B**). All data are presented as mean ± SD. *n* = 8. ** *p* < 0.01 and **** *p* < 0.0001 vs. Cont group. ^#^ *p* < 0.05, and ^####^ *p* < 0.001, vs. Ade group.

To determine the effect of CC on AD-induced fibrotic responses, we assessed two ECM proteins, including type IV collagen and fibronectin (FN), using RT-PCR. Compared with those in the Cont group, the mRNA expression levels of type IV collagen and FN were significantly increased in the Ade group (*p* < 0.01 and *p* < 0.001, respectively). However, the mRNA expression levels were decreased in the Ade+CC group compared with those in the Ade group (Figure 5A,B, *p* < 0.05, *p* < 0.001, respectively). Our results suggest that CC attenuated ECM overproduction and renal fibrosis in CKD mice.

### 2.5. CC Attenuates the Expression of Profibrotic Genes in AD-Induced Renal Injury Mice

**Figure 6:**
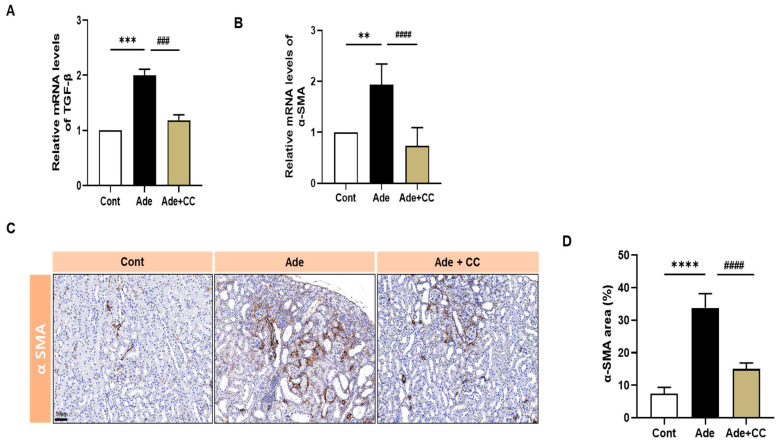
CC reduces the upregulation of TGF-β1 and α-SMA expression in AD-induced renal injury mice. qRT-PCR results of the mRNA levels of TGF-β1 (**A**) and α-SMA (**B**). IHC staining showing the expression of α-SMA (**C**). ImageJ was used for quantification of the α-SMA area (**D**). All data are expressed as mean ± SD. *n* = 8. ** *p* < 0.01, *** *p* < 0.001, and **** *p* < 0.0001, vs. Cont group. ^###^ *p* < 0.001, and ^####^ *p* < 0.0001 vs. Ade group.

To define the effect of CC in regulating fibrosis, we assessed the expression levels of fibrosis-related genes, including transforming growth factor (TGF)-β and α-smooth muscle actin (SMA), using qRT-PCR. A significant increase in TGF-β and α-SMA expression levels in the Ade group compared with those in the Cont group was observed (Figure 6A,B, *p* < 0.001 and *p* < 0.01, respectively). However, the Ade+CC group exhibited a reduction in these parameters related to renal fibrosis (*p* < 0.001 for both). Furthermore, we assessed α-SMA by immunohistochemistry (IHC) staining. Tubulointerstitial fibrosis in the Ade group mice was significantly higher than that in the Cont group (*p* < 0.0001). However, compared with that in the Ade group, the tubulointerstitial fibrosis in mice in the Ade+CC group was also decreased (Figure 6C,D, *p* < 0.0001). These results suggest that CC attenuated profibrotic gene expression and tubulointerstitial fibrosis in CKD mice.

## 3. Discussion

CKD is a progressive and irreversible disease, increasingly becoming more common and a critical health challenge with no effective therapy. Thus, many researchers have investigated the pathogenesis of CKD, its underlying mechanisms, and the development of effective and new treatments. In humans, AD causes metabolic abnormalities that mimic CKD. Accumulating evidence has shown that CKD is induced in mice fed an AD diet, a well-established model for exploring renal injury [8,21]. After feeding, AD is metabolized to 2,8-dihydroxyadenine and deposited and crystallized in the proximal renal tubules owing to renal injury, leading to the accumulation of urine and inflammation of the tubule, causing tubulointerstitial fibrosis [9]. Our results indicate that mice fed with AD exhibited significant weight loss and renal dysfunction owing to markedly increased levels of BUN and SCr. In addition, our findings showed that the kidneys of AD-fed mice exhibited significant tubular and interstitial injuries, including tubular atrophy, dilation, interstitial inflammation, and fibrosis. Consistent with previous studies [9,10,21], these findings provide sufficient evidence of renal damage in AD-fed mice. In the present study, we observed that CC treatment effectively reduced the increase in BUN and SCr levels and histological renal morphology in AD-induced CKD mice. These findings suggest that CC is a potential inhibitor of renal injury.

Furthermore, evidence indicates that the progression of CKD involves inflammation and fibrosis. Accumulating evidence further indicates that the increase in inflammatory cytokines, including TNF-α, IL-1β, and IL-6, facilitates the progression of CKD [22,23]. Similarly, AD feeding increased the expression of renal inflammatory factors, such as IL-6 and IL-1β [24]. In addition, MCP-1 expression was associated with increased COX-2 [25]. MCP-1, an inflammatory cytokine, increases the proliferation, infiltration, and production of several cytokines and chemokines in inflammatory cells [24,26]. ICAM-1 is a chemotactic factor that enables macrophages to maintain an inflammatory response. Activated ICAM-1 recruit macrophages. Thus, ICAM-1 is an essential factor for macrophage infiltration into the renal tissue. Moreover, F4/80 is commonly recognized as an indicator of macrophage infiltration. Thus, the increased expression of F4/80 in the kidneys represented a vigorous inflammatory process and has been well-documented in kidney injuries [27]. In this study, CC inhibited the increased expression of MCP-1, IL-6, IL-1β, COX-2, ICAM-1, and F4/80 in AD-induced kidney injury. These findings suggest that CC exerts anti-inflammatory effects on AD-induced renal injury.

Furthermore, the development of CKD is associated with tubulointerstitial fibrosis [28]. TGF-β1 has a crucial role in the renal fibrosis process [29] and induces epithelial-to-mesenchymal transition (EMT) [30,31]. Myofibroblasts produce inflammatory cytokines and collagenous matrix, leading to renal fibrosis. The renal tubules with EMT show an upregulated expression of α-SMA and ECM proteins (FN and type IV collagen) [9]. TGF-β1 promotes an increased ECM production, including FN and type IV collagen [32]. Several studies reported that feeding mice with an AD diet increases the expression of TGF-β1 and ECM proteins, such as type IV collagen and FN, resulting in renal fibrosis [5,32]. In this study, AD treatment significantly increased TGF-β1, type IV collagen, FN, and α-SMA expression in the kidneys, which were significantly suppressed by CC treatment. Our results suggest that CC may possess antifibrotic effects in patients with CKD.

Epidemiological evidence showed that consuming a diet containing enough vegetables, medicinal plants, or their derived flavonoids can delay the progression of CKD possibly be due to the beneficial effect of several components and other phytochemicals that act synergistically. Hence, we investigated whether CC can be used as an alternative target to alleviate CKD in AD-fed mice. Accumulating evidence indicates that CC and CC-derived compounds have beneficial effects [16]. Similar to our findings, previous studies have demonstrated that CC contains caffeic acid, chlorogenic acid, rutin, cynarine, 3,4-dicaffeoylquinic acid, and 3,5-dicaffeoyl-4-succinoylquinic acid [14,15]. Rutin has numerous beneficial health effects, including anti-inflammatory and antifibrotic effects in CKD [16,17,33]. Caffeic acid exhibits pharmacological activities, including antioxidant and antifibrotic effects, and reduces renal tubulointerstitial fibrosis [34,35]. Chlorogenic acid attenuates renal fibrosis [36]. Furthermore, 3,4-dicaffeoylquinic acid possesses antioxidant and anti-inflammatory properties [37]. Therefore, we hypothesized that the CC extract, containing several bioactive compounds, would exert an inhibitory effect on AD-induced renal injury. Herein, the CC extract showed protective effects in improving renal function in AD-induced CKD by suppressing inflammatory factors and fibrosis, suggesting that the extract might be used as a potential renoprotective treatment or functional food supplement to manage CKD.

## 4. Materials and Methods

### 4.1. Materials and Reagents

*Chrysanthemum. coronarium* L. leaves were purchased from a local market (Jeonju, Republic of Korea). AD, methanol (MeOH), and ethanol were obtained from Sigma-Aldrich (St. Louis, MO, USA). Radioimmunoprecipitation assay buffer was purchased from Beyotime (Los Altos, CA, USA). BCA Protein Assay Kit was purchased from Thermo Fisher Scientific (Waltham, MA, USA). SDS-PAGE gels and polyvinylidene fluoride membranes (PVDF) were obtained from Millipore (Burlington, MA, USA). TRIzol reagent and SYBR Green PCR Master Mix Kit were obtained from Invitrogen (Waltham, CA, USA). ICAM-1, COX-2, α-SMA, and β-actin antibodies were purchased from Santa Cruz Biotechnology (Dallas, TX, USA). Goat anti-rabbit secondary antibodies were obtained from Cell Signaling Technology (Beverly, MA, USA). iScript cDNA synthesis kit was purchased from Bio-Rad (Hercules, CA, USA). BUN, SCr, calcium, and phosphorous kits were purchased from Chema Diagnostica Di Fiore Marco (Monsano, Italy).

### 4.2. Preparation and Analysis of CC

The dried CC (1 kg) was crushed and extracted twice with 10 L of 50% ethanol for 24 h. After filtration, the extracts were evaporated by rotary evaporator and freeze-dried at −70 °C. The extraction yield was 8% (*w*/*w*). All experiments on *Chrysanthemum coronarium* L. leaves were performed in accordance with the institutional, national, and international guidelines and legislation. CC (10 mg) was dissolved in 50% MeOH (10 mL), and the chemical profile of the extract was analyzed using UPLC-tunable UV (TUV) (Waters, Milford, MA, USA) with an Acquity UPLC BEH C18 column (100 × 2.1 mm, 1.7 μm; Waters). The eluted compounds were analyzed by Q-TOF/MS (Waters) in the negative electrospray ionization mode under optimized conditions as follows: a scan range of 50–1500 *m*/*z*, a scan time of 0.2 s, a capillary voltage of 2.5 kV, a sampling cone voltage of 40 V, a desolvation flow rate of 900 L/h, a desolvation temperature of 400 °C, source temperature of 100 °C, and sample injection volume of 1 μL. MS/MS spectra were acquired under collision energy ramp (10–30 eV). The major compounds were identified based on the online database UNIFI 1.8.2 (Waters) and other online databases, such as ChemSpider, Traditional Chinese Medicine database, and METLIN [16].

### 4.3. Animals

C57BL/6 male mice were obtained from Oriental Biotechnology (Daejeon, Republic of Korea). The mice were maintained in controlled rooms with a temperature of 22 ± 2 °C and humidity of 50 ± 60% under a 12:12 h light/dark cycle and had access to food and water ad libitum. The animal experiments were approved by the Institutional Animal Care and Use Committee of the Korea Food Research Institute (KFRI: KFRI-M- 22001) and performed in conformance with the institutional guidelines established by the Committee.

### 4.4. Experimental Procedure

Thirty mice weighing 20–22 g were randomly assigned into three experimental groups, namely called control (Cont, *n* = 10), AD (Ade, *n* = 10), and AD with CC (Ade+CC, *n* = 10). The control group was fed normal chow diet. CKD was induced by feeding mice with an AD diet (0.25% *w*/*w*). The Ade group was fed an AD diet supplemented with saline. The Ade+CC group was fed an AD diet supplemented with CC. CC was orally administrated once daily at 100 mg/kg/day. The Cont and AD groups were administered normal saline. Body weights were measured weekly. We tried to minimize animal suffering and used the appropriate number of mice to obtain reliable data. After 4 weeks of feeding, all the mice were euthanized with 1.5% isoflurane (BKPharma Corp., Goyang-si, Republic of Korea). Blood was collected from each mouse, and serum was obtained by centrifugation at 12,000 rpm and 4 °C for 12 min. The kidneys were excised and fixed. Serum and renal tissues were kept at −80 °C.

### 4.5. Biochemical Parameters

Common serum biochemical parameters, including BUN, SCr, calcium, and phosphorous, were measured using a biochemical kit, following the manufacturer’s instructions.

### 4.6. Histopathological Analysis

The fixed kidneys were dehydrated and paraffin-embedded. The samples were then sectioned at 4 µm thickness, dewaxed, and rehydrated. Slides were stained with H&E, PAS, and MT to analyze inflammation, fibrosis, atrophy, and dilation [38]. The slides were analyzed under a panoramic microscope (3D HISTECH Panoramic 250, Budapest, Hungary).

### 4.7. IHC

The expression of α-SMA was analyzed by IHC staining. Sectioned slides were blocked with 5% BSA /PBS at 25 °C for 20 min and then incubated with the anti-ɑ-SMA (1:500) at 4 °C overnight. After incubation with secondary antibodies, the slides were visualized using 3,3′-diaminobenzidine (Dako, CA, USA). The sections were observed using a panoramic microscope (3D HISTECH Panoramic 250, Hungary), and analyzed by ImageJ 1.53a software [39].

### 4.8. RNA Isolation and qRT-PCR

RNA was isolated from the kidney tissues (100 mg) using an RNeasy RNA isolation kit. The concentration of the extracted RNA was analyzed by a NanoDrop Spectrophotometer (Thermo Scientific, Waltham, MA, USA). Subsequently, the RNA was reverse-transcribed to cDNA by an iScript cDNA synthesis kit with random primers. Finally, the synthesized cDNA was used for qRT-PCR using the SYBR Green PCR Master Mix Kit. The expression of the housekeeping gene glyceraldehyde 3-phosphate dehydrogenase served as the endogenous control. The actual primer sequences are showed in Table 1.

### 4.9. Western Blotting Analysis

Western blotting was performed as previously described [40]. The Kidney tissues were homogenized in a radioimmunoprecipitation assay buffer. The protein concentration of kidney samples was determined using the BCA protein assay kit. Similarly, 20 µg of tissue lysates was loaded onto SDS-PAGE gels, separated, and transferred onto PVDF membranes. Next, the membranes were applied with 5% skimmed milk and incubated with ICAM-1, COX-2, and β-actin antibodies. Next, the membranes were incubated with anti-rabbit secondary antibodies. Finally, signals were visualized using an image analyzer (ChemiDoc^TM^ XRS+ System; Bio-Rad Laboratories) and densitometry was performed using ImageJ (NIH).

### 4.10. Statistical Analysis

Results are shown as the mean ± standard derivation (SD) and analyzed using GraphPad Prism software version 9.0 (Inc., La Jolla, CA, USA). One-way analysis of variance, together with Tukey’s multiple comparison test, was carried out to detect statistical differences. Significant differences were regarded at *p* < 0.05.

## 5. Conclusions

CC possesses various beneficial effects; however, its protective effect on AD-induced chronic renal injury remained unclear. In this study, we confirmed that CC could improve renal function and protect against AD-induced chronic renal injury. In addition, we found that the mechanism of renoprotection may be related to its anti-inflammatory and antifibrotic activities. Nevertheless, this study had some limitations. First, we focused on inflammation and fibrosis in the kidneys. Hence, we assessed only the associated parameters. Second, we did not determine the therapeutic effects of the various CC-derived compounds. Therefore, further studies are needed to understand the mechanistic signaling pathways involved in the effects of CC and CC-derived components.

## Figures and Tables

**Table 1:** List of primers used in the study.

Gene	Primer Sequence (5′-3′)	Tm (°C)
MCP-1	F: GCCTGCTGTTCACAGTTGC	59.1
	R: CAGGTGAGTGGGGCGTTA	58.0
IL-6	F: TGAGAGTAGTGAGGAACAAG	52.8
	R: CGCAGAATGAGATGAGTTG	53.0
IL-1β	F: TGAGCTCGCCAGTGAAATGAT	59.1
	R: TCCATGGCCACAACAACTGA	58.8
F4/80	F: CCTGGACGAATCCTGTGAAG	57.0
	R: GGTGGGACCACAGAGAGTTG	59.0
Type IV collagen	F: TTAAAGGACTCCAGGGACCAC	58.0
	R: CCCACTGAGCCTGTCACAC	59.3
Fibronectin	F: CCCTATCTCTGATACCGTTGTCC	58.8
	R: TGCCGCAACTACTGTGATTCGG	62.4
TGFβ-1	F: TCAGACATTCGGGAAGCAGT	58.0
	R: ACGCCAGGAATTGTTGCTAT	56.9
α-SMA	F: GCCCAGAGCAAGAGAGG	55.6
	R: TGTCAGCAGTGTCGGATG	56.1
GAPDH	F: AAATGGTGAAGGTCGGTGTG	60.0
	R: TGAAGGGGTCGTTGATGG	60.0

## Data Availability

Data are contained within the article.
